# Signaling pathways activated and regulated by stem cell-derived exosome therapy

**DOI:** 10.1186/s13578-024-01277-7

**Published:** 2024-08-20

**Authors:** Ding Li, Danni Li, Zhao Wang, Jiaojiao Li, Khawar Ali Shahzad, Yanhong Wang, Fei Tan

**Affiliations:** 1 Department of ORL-HNS, Shanghai Fourth People’s Hospital, School of Medicine, Tongji University, Shanghai, China; 2 Plasma Medicine and Surgical Implants Center, Tongji University, Shanghai, China; 3 The Royal College of Surgeons in Ireland, Dublin, Ireland; 4 The Royal College of Surgeons of England, London, UK

**Keywords:** Exosome, Stem cell, Pathway, Signaling cascade, Surgery

## Abstract

Stem cell-derived exosomes exert comparable therapeutic effects to those of their parental stem cells without causing immunogenic, tumorigenic, and ethical disadvantages. Their therapeutic advantages are manifested in the management of a broad spectrum of diseases, and their dosing versatility are exemplified by systemic administration and local delivery. Furthermore, the activation and regulation of various signaling cascades have provided foundation for the claimed curative effects of exosomal therapy. Unlike other relevant reviews focusing on the upstream aspects (e.g., yield, isolation, modification), and downstream aspects (e.g. phenotypic changes, tissue response, cellular behavior) of stem cell-derived exosome therapy, this unique review endeavors to focus on various affected signaling pathways. After meticulous dissection of relevant literature from the past five years, we present this comprehensive, up-to-date, disease-specific, and pathway-oriented review. Exosomes sourced from various types of stem cells can regulate major signaling pathways (e.g., the PTEN/PI3K/Akt/mTOR, NF-κB, TGF-β, HIF-1α, Wnt, MAPK, JAK-STAT, Hippo, and Notch signaling cascades) and minor pathways during the treatment of numerous diseases encountered in orthopedic surgery, neurosurgery, cardiothoracic surgery, plastic surgery, general surgery, and other specialties. We provide a novel perspective in future exosome research through bridging the gap between signaling pathways and surgical indications when designing further preclinical studies and clinical trials.

## Introduction

### Stem cell therapy

Stem cells possess the unique abilities to self-renew and develop into differentiated cells. Stem cell therapy has therefore been explored in regenerative medicine for the treatment of various diseases. Although stem cell transplantation has demonstrated clinical value in certain conditions including immune disorders, hematological dysfunctions, and tissue injuries, the translation of the clinical trials into bedside practice remains unsatisfactory [1]. This is likely due to the biosafety concerns of stem cell therapy, such as infusion toxicity, immune response, oncological complication, and ethical issue [2]. Alternatively, stem cell-derived exosome therapy may provide similar therapeutic benefits without the aforementioned drawbacks with regard to stem cell therapy [3].

### Stem cell-derived exosome therapy

Stem cells partly exert their beneficial effects through paracrine actions such as release of exosomes [4]. Exosomes are lipid bi-layered membrane-bound extracellular vesicles measuring approximately 50–120 nm. Just like other cells in the body, stem cells deliver exosomes to communicate with each other and/or non-stem cells [5]. Exosomes could be considered miniature versions of their donor cells because exosomes from a certain cell type provide unique sets of soluble secretomes. As a result, stem cell-derived exosomes (SC-Exo) inherit similar therapeutic effects (e.g., anti-inflammation and tissue regeneration) from their parental cell of origin [6]. In contrast to stem cells, SC-Exo has minimal tumorigenic and immunogenic complications, lack ethical issues, and are amenable to versatile delivery routes [7].

### Biogenesis and composition of exosomes

**Fig. 1:**
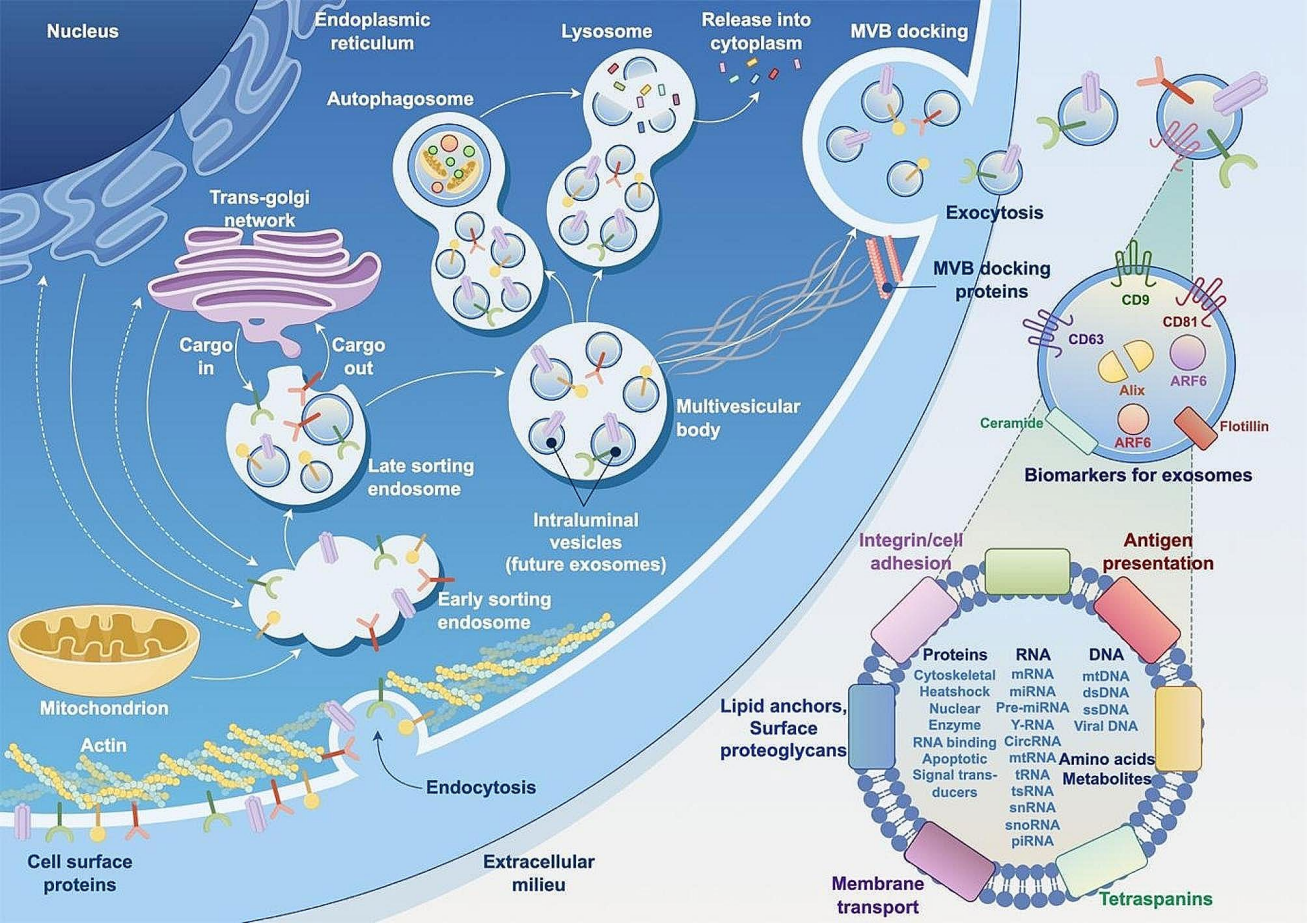
Biogenesis and composition of exosomes. Figure generated using FigDraw online platform (www.figdraw.com) with an export ID of STIATf0909

Exosome biogenesis typically follows an endosomal route (Fig. 1) [8]. The early sorting endosomes are formed by endocytosis of the bioactive substances in the form of cell membrane invagination. Then, the late sorting endosomes are formed by incorporating cargoes from the Golgi network. The subsequently produced multivesicular bodies (MVBs) after a second indentation attach to MVB docking proteins. Finally, the docked MVBs fuse with the cell membrane, releasing the contained exosomes in the form of exocytosis. These exosomes could be identified according to their surface biomarkers. Irrespective of the type of donor cells, the released exosomes carry numerous functional cargoes, including proteins, nucleic acids, metabolites, glycoconjugates, lipids, and other bioactive substances (Fig. 1) [9, 10].

### Preclinical applications of exosomes

**Fig. 2:**
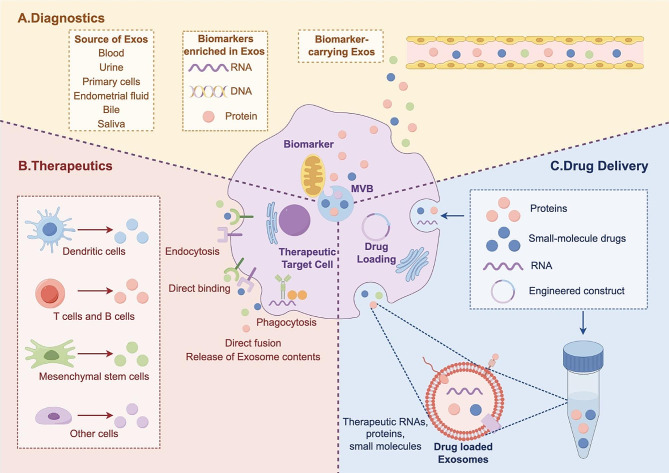
Major preclinical and clinical applications of exosomes: diagnostic applications, therapeutic approaches, and drug delivery. Figure generated using FigDraw online platform (www.figdraw.com) with an export ID of IORUY101fa

More than 300 preclinical studies have been conducted utilizing exosomes. These applications can be broadly categorized into diagnostic analyses, therapeutic applications and drug delivery [11] (Fig. 2). Firstly, body fluid-derived exosomes are a highly stable reservoir of disease biomarkers, assisting liquid biopsy in various clinical settings such as cardiovascular diseases, perinatal disorders, and cancers [12, 13]. Secondly, almost all types of human cells can generate exosomes, including, but are not limited to stem cells, dendritic cells (DCs), immune cells, and even tumor cells [14]. Some preclinical trials have demonstrated the effectiveness and safety of DC-derived exosomes (Dex)-based immunotherapy for cancers [15]. Lastly, exosomes may exert as an ideal carrier to deliver drugs due to their advantages in stability, non-immunogenicity, and targeting recipient cells [16]. For example, doxorubicin could be loaded in Dex using electroporation for the treatment of breast cancer [17].

### Signaling pathways activated and regulated by SC-Exo therapy

In the context of therapeutic applications of stem cell-derived exosomes, their therapeutic effects for various diseases are achieved through diverse changes at tissue level (e.g., tissue regeneration, immunomodulation, and anti-inflammation), cellular level (e.g., promotion of cell proliferation, migration and differentiation, and inhibition of cell death and oxidative stress), and molecular level (activation and regulation of signaling pathways such as the PTEN/PI3K/Akt/mTOR, NF-κB, TGF-β, HIF-1α, Wnt, MAPK, JAK-STAT, Hippo, and Notch cascades). This hierarchical magnification of multi-level benefits supports SC-Exo therapy as a potent and versatile alternative, and even superior to stem cell-based therapy.

Unlike other reviews on SC-Exo therapy, the uniqueness of this review is threefold. Firstly, the current work focuses on the most upstream of the chain of events, i.e., molecular mechanism. Secondly, it then relates these molecular events with the most downstream of therapeutic chain, i.e., clinical indication. Finally, this review dissects relevant publications from the last five years covering exosomes delivered both systemically and locally. Systemic administration (e.g., through venous injection) and local delivery (e.g., assisted by biomaterials) of SC-Exo each has advantages and disadvantages, highlighting the versatility of SC-Exo therapy.

## The PTEN/PI3K/Akt/mTOR pathway regulated by SC-Exo therapy

**Fig. 3:**
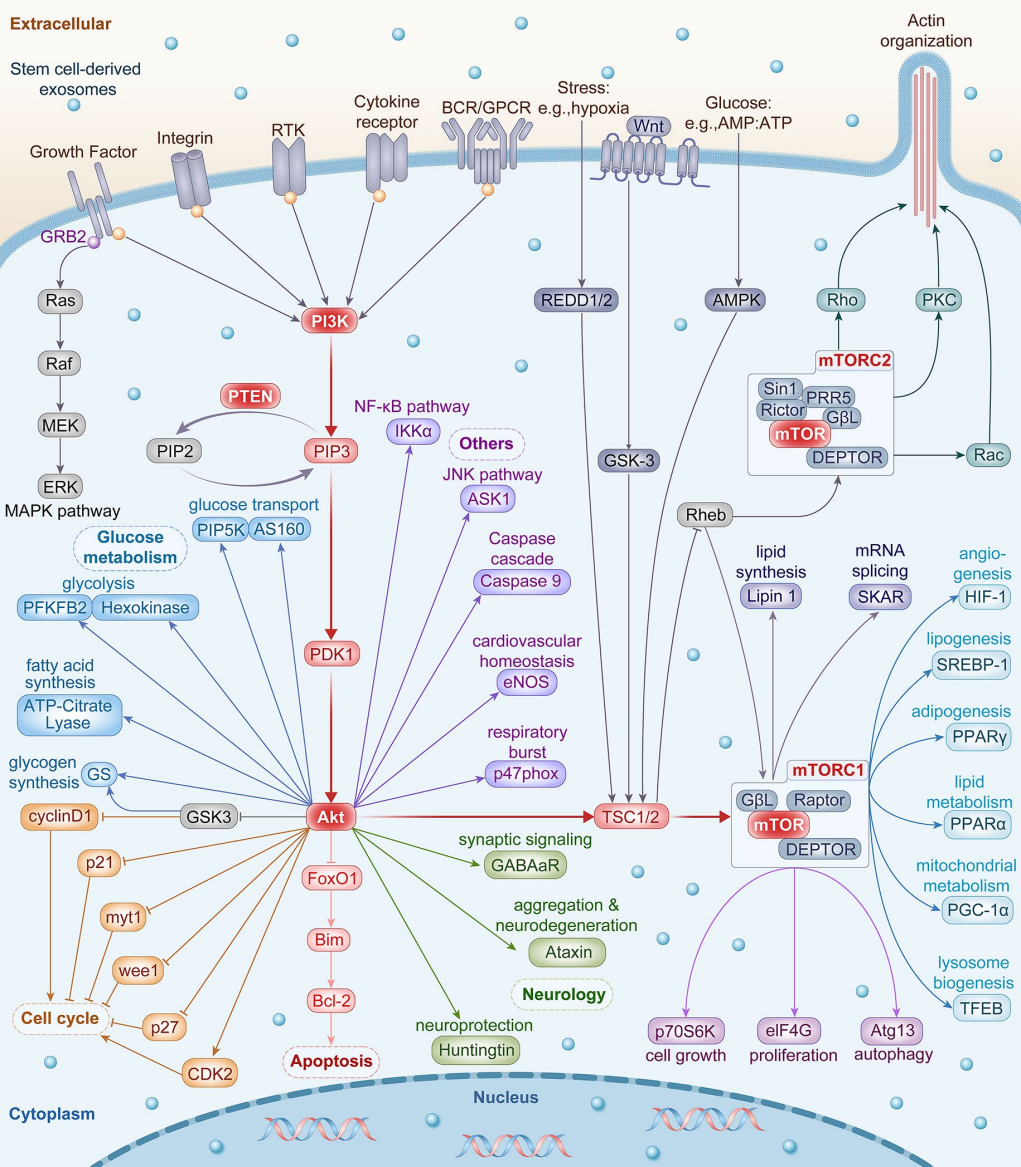
Schematic diagram of the PTEN/PI3K/Akt/mTOR signaling pathway. Figure generated using professional drawing service provided by FigDraw (www.figdraw.com)

The phosphatase and tensin homolog (PTEN) / phosphoinositide 3-kinase (PI3K) / protein kinase B (Akt) / mammalian target of rapamycin (mTOR) pathway is one of the most important signaling pathways that plays a crucial role not only in physiological cellular functions (e.g., cell cycle, proliferation, survival, metabolism and motility) but also in cancer development, progression, and treatment (e.g., breast cancer, hepatocellular carcinoma, prostate cancer, and lung cancer) [18–22]. As a lipid phosphatase, PTEN is the principle negative regulator for the pro-survival and oncogenic PI3K/Akt/mTOR pathway by dephosphorylating PIP3 to PIP2 [23]. Upstream activation of PI3K is initiated by a variety of extracellular stimuli such as growth factors and nutritional changes (Fig. 3). This activation is achieved through transmembrane structures such as integrins, cytokine receptors, Toll-like receptors (TLRs), receptor tyrosine kinases (RTKs), B-cell antigen receptors (BCRs), and G protein-coupled receptors (GPCRs) [24]. Subsequently, the multifunctional Akt wires over one hundred molecular substrates, playing a pivotal role in cell cycle, apoptosis, and glucose metabolism [25]. In addition, the disruption of Akt network is associated with cancer, diabetes, inflammatory and autoimmune disorders, and cardiovascular and neurological diseases [26]. Finally, as one of the most crucial downstream nodes of the PI3K/Akt cascades, mTOR is at the crossroads of nutrition, growth, ageing and disease. The functions of mTOR include, but are not limited to, activation of protein synthesis, biomass accumulation, and repression of catabolism and autophagy [27] (Fig. 3). Most importantly, since no pathways exist independently, there is extensive crosstalk between PTEN/PI3K/Akt/mTOR pathway and other signaling pathways such as MAPK pathway, NF-κB pathway, JNK pathway, and Wnt pathway [28]. As a matter of fact, PTEN/PI3K/Akt/mTOR pathway is the most popularly studied signaling cascade during mechanistic exploration of SC-Exo therapy (Table 1).

**Table 1:** Major signaling pathways influenced by stem cell-derived exosome-based therapy

Target disease	Source of exosome	Modification of exosome	Delivery modality	Essential findings	Refs
**PTEN/PI3K/Akt/mTOR pathway**					
fracture	BM MSC	DMOG-stimulated	systemic	enhanced bone regeneration through angiogenesis in rats	[29]
fracture	BM MSC	N/A	local	exosomal miR-130a mediated pro-angiogenic activity in bone remodeling in rats	[30]
fracture	UC MSC	N/A	local	exosomal miR-23a-3p achieved vascularized bone regeneration in rats with skull defect	[31]
OA	IPFP MSC	N/A	systemic	exosomal miR-100-5p protected articular cartilage and ameliorated gait abnormalities in mice	[32]
OA	BM MSC	hypoxia precondition	local	exosomal miR-205-5p promoted cartilage regeneration in mice	[33]
IDD	BM MSC	N/A	systemic	exosomal miR-21 alleviated nucleus pulposus apoptosis and IVD degeneration	[34]
IDD	CESC	Sphk2-engineered	local	regulated autophagy/senescence and prevented disc degeneration in rats	[35]
RA	OE MSC	N/A	local	exosomal PD-L1 relieved synovial inflammation and joint destruction by suppressing Tfh cell polarization in mice	[182]
SCI	BM MSC	hypoxia precondition	systemic	exosomal miR-216a-5p promoted functional behavioral recovery by shifting microglial M1/M2 polarization in mice	[36]
SCI	NSC	FTY720-loaded	systemic	ameliorated hindlimb function and reduced inflammation by downregulating Bax and aquaporin-4 and upregulating claudin-5 and Bcl-2 in rats	[37]
SCI	USC	N/A	local	exosomal ANGPTL3 enhanced spinal cord neurological functional recovery by promoting angiogenesis in mice	[38]
SCI	BM MSC	N/A	local	decreased inflammation, enhanced local NSCs recruitment, and promoted neuronal regeneration, resulting in functional recovery in mice	[39]
ischemic stroke	NSC	TSG101 oe	systemic	protected brain via anti-inflammatory activities, DNA damage pathway inhibition, and growth/trophic factor induction in rats	[40]
wound healing	BM MSC	deferoxamine -stimulated	systemic	exosomal miR-126 accelerated cutaneous wound healing by promoting angiogenesis in rats with streptozotocin-induced diabetic wound	[42]
wound healing	adipose MSC	N/A	systemic	promoted fibroblast proliferation and migration, and optimized collagen deposition in mice	[43]
wound healing	BM MSC	N/A	local	adjusted wound inflammation microenvironment, promoted neovascularization, and accelerated wound healing in type I diabetic rats	[44]
wound healing	adipose MSC	N/A	local	promoted diabetic wound healing by optimizing cellular functions and relieving oxidative stress in mice	[45]
liver fibrosis	BM MSC	circDIDO1 oe	systemic	exosomal miR-141-3p could suppress hepatic stellate cell activation in human liver fibrosis	[46]
IHD	BM MSC	N/A	systemic	exosomal miR-144 ameliorated cardiomyocyte apoptosis under hypoxic conditions	[48]
microtia	adipose MSC	miR-23a-3p oe	local	promote chondrocyte survival and proliferation, attenuated cell apoptosis, and stimulated new cartilage regeneration	[49]
**NF-κB pathway**					
tendon injury	SHED	young exo	local	reduced senescent cells and ectopic bone formation, thereby rescuing endogenous tendon regeneration in naturally ageing mice	[56]
RA	gingival MSC	N/A	systemic	immunosuppressive, and reduced bone erosion of collagen-induced arthritis in mice via inhibiting IL-17RA-Act1-TRAF6-NF-κB axis	[57]
growth plate injury	BM MSC	N/A	local	facilitated cartilage matrix formation, and boosted repair of growth plates and reduced bone bridge formation in rats by polarizing macrophages	[58]
TBI	adipose MSC	N/A	systemic	specifically entered microglia/macrophages and suppressed their activation, thereby inhibiting inflammation and facilitating functional recovery in rats	[59]
TBI	NSC	N/A	local	attenuated oxidative stress via TLR4/NF-κB/IL-1β, reactive gliosis, lesion volume, and increased neurogenesis via bio-bridge mechanism in rats	[60]
SCI	NSC	IGF-1 stimulated	systemic	IGF exosomal miR-219a-2-3p promoted neuroprotective effects and reduced neuroinflammation in rats by inhibiting YY1	[61]
SCI	BM MSC	N/A	local	decreased inflammation, enhanced local NSCs recruitment, and promoted neuronal regeneration, resulting in functional recovery in mice	[39]
SNI	BM MSC	N/A	local	enhanced myelinated axonal regeneration, relieved inflammatory pain, thereby ameliorating muscle atrophy and promoting functions in diabetic rats	[62]
ischemic stroke	UC MSC	N/A	systemic	exosomal miR-146a-5p reduced microglia-mediated neuroinflammation via suppression of IRAK1/TRAF6 in mice	[63]
MS	BM MSC	N/A	systemic	improved cognitive function, promoted remyelination, reduce neuroinflammation, and blocked TLR2/IRAK1/NFκB signaling in cuprizone mice	[64]
wound healing	adipose MSC	N/A	local	accelerated wound closure via metabolic pathways, tight junction, NF-κB pathway in rats	[65]
wound healing	placental MSC	miR-146a-engineered	local	promoted wound healing with anti-inflammation, collagen deposition, and neovascularization in mice by targeting IRAK1	[66]
chronic endometritis	BM MSC	IL-1β-activated	local	exerted excellent effects on anti-inflammation and endometrial regeneration in rats	[67]
**TGF-β pathway**					
fracture	BM MSC	N/A	local	induced rapid bone regeneration via Bmpr2/Acvr2b competitive receptor-activated Smad pathway in rats	[76]
tendon injury	adipose MSC	N/A	local	inhibited early inflammatory reaction and promoted tendon healing by activating both SMAD1/5/9 & SMAD2/3	[77]
SCI	BM MSC	N/A	systemic	promoted M2 macrophage polarization, upregulated TGF-β, and reduced BSCB leakage in rats	[78]
wound healing	UC MSC	N/A	systemic	suppressed myofibroblast differentiation during wound healing in mice by inhibiting TGF-β/Smad2; miR-21, -23a, -125b, -145 responsible for preventing scar formation	[82]
wound healing	UC MSC	N/A	systemic	suppressed dermal fibroblasts-myofibroblasts transition by inhibiting TGF-β/Smad2/3	[81]
wound healing	UC MSC	N/A	local	accelerated wound closure rate, and upregulated CD31 and Ki67, VEGF and TGFβ-1 in rats	[83]
scleroderma	UC MSC	N/A	systemic	ameliorated dermal fibrosis by attenuating myofibroblast activation and collagen deposition	[84]
**HIF-1α pathway**					
fracture	UC MSC	hypoxia precondition	systemic	hypoxia enhanced exo production, and exosomal miR-126 promoted fracture healing in mice	[92]
fracture	UC MSC	N/A	local	enhanced fracture healing through HIF-1α-mediated angiogenesis in rats with stabilized fracture	[94]
SCI	UC MSC	hypoxia precondition	local	promoted angiogenesis and locomotor function in rats	[95]
wound healing	adipose MSC	N/A	systemic	promoted wound healing through accelerated keratinocyte proliferation & migration	[96]
wound healing	adipose MSC	hypoxia precondition	local	exosomal circ-Snhg11 enhanced wound healing and angiogenesis via miR-144-3p/NFE2L2/HIF-1α in mice	[97]
wound healing	epidermal SC	VH298-loaded	local	promoted wound healing by locally enhancing blood supply and angiogenesis in diabetic mice	[98]
MI	ASC	Tβ4-engineered	local	exosomal miR-17-5p promoted coronary collateralization in periphery of myocardial infarcted area, and cardiac repair	[99]
**Wnt pathway**					
fracture	BM MSC	N/A	systemic	exosomal miR-136-5p promoted fracture healing by targeting LRP4 in mice	[109]
OA	BM MSC	miR-92a-3p	systemic	enhanced chondrogenesis and suppressed cartilage degradation in mice; miR-92a-3p as a Wnt inhibitor and DMOAD	[110]
OA	synovial MSC	miR-140-5p oe	systemic	exosomal Wnt5a & Wnt 5b enhanced cartilage tissue regeneration and reduced cartilage matrix loss in rats by activating YAP	[111]
ischemic stroke	BM MSC	Zeb2/Axin2 enriched	systemic	promoted functional recovery by enhancing neurogenesis and neural plasticity via SOX10, Wnt/β-catenin, and endothelin-3/EDNRB pathways	[112]
wound healing	adipose MSC	MALAT1	systemic	mediated H_2_O_2_-induced wound healing by targeting miR-124	[113]
alopecia	NSC	N/A	systemic	exosomal miR-100 promoted hair follicle growth in depilation-induced mice	[114]
**MAPK pathway**					
TBI	adipose MSC	N/A	systemic	specifically entered microglia/macrophages and suppressed their activation, thereby inhibiting inflammation and facilitating functional recovery in rats	[59]
SCI	placental MSC	N/A	systemic	promoted NSC proliferation by MEK/ERK/CREB, and activated endogenous neurogenesis and improved locomotor activity and bladder dysfunction	[120]
SNI	BM MSC	N/A	local	enhanced myelinated axonal regeneration, relieved inflammatory pain, thereby ameliorating muscle atrophy and promoting functions in diabetic rats	[62]
ischemic stroke	NSC	RGD targeting ligands	systemic	targeted ischemic brain regions and suppressed postischemia inflammatory response	[121]
AKI	iPSC	N/A	systemic	corrected serum creatinine level, tubular necrosis, apoptosis, inflammatory cytokine production, and oxidative stress in mice with renal IRI	[123]
**JAK-STAT pathway**					
TBI	BM MSC	N/A	systemic	reduced cortical tissue apoptosis and inhibited neuroinflammation, possibly by exosomal miR-181b via IL-10/STAT3	[128]
PD	BM MSC	N/A	systemic	exosomal TSG-6 attenuated MPP^+^-induced neurotoxicity via STAT3/miR-7/NEDD4 axis	[129]
myocardial IRI	NSC	N/A	systemic	reduced infarct size while delaying cardiomyocyte mitochondrial permeability transition pore opening in mice via JAK1/2 and gp130	[130]
**Hippo pathway**					
fracture	PDLSC	N/A	local	enhanced osteoinductivity and osteogenesis via YAP/TAZ	[135]
osteoporosis	UC MSC	N/A	systemic	exosomal miR-1263 prevented apoptosis in disuse osteoporosis rats by targeting Mob1	[136]
POI	UC MSC	N/A	systemic	restored ovarian function-related hormone levels and the number of ovarian follicles, and improved the reproductive ability of POI mice	[137]
**Notch pathway**					
fracture	UC MSC	N/A	local	accelerated bone repair by enhancing angiogenesis via miR-21/NOTCH1/DLL4 in rats with cranial defect	[142]
mechanical allodynia	BM MSC	N/A	systemic	exosomal miR-150-5p reduced apoptosis and inflammation in spinal dorsal horn by targeting NOTCH2 in microglia, thereby alleviating disease in rats	[143]

(AKI = acute kidney injury, akt = protein kinase B, ANGPTL = angiopoietin-like protein, ASC = artificial stem cell, BM = bone marrow, BSCB = blood-spinal cord barrier, CESC = cartilage endplate stem cell, CREB = cAMP response element binding, DLL = Delta-like protein, DMOAD = disease-modifying OA drug, DMOG = dimethyloxaloylglycine, EDNRB = endothelin receptor type B, ERK = extracellular signal-regulated kinase, exo = exosome, HIF = hypoxia-inducible factor, IDD = intervertebral disc degeneration, IGF = insulin-like growth factor, IHD = ischemic heart disease, IL = interleukin, iPSC = induced pluripotent stem cell, IPFP = infrapatellar fat pad, IRAK = interleukin-1 receptor-associated kinase, IRI = ischemia reperfusion injury, IVD = intervertebral disc, JAK = Janus kinase, LRP = lipoprotein receptor related protein, MALAT = metastasis associated lung adenocarcinoma transcript, MAPK = mitogen-activated protein kinase, MEK = mitogen-activated protein kinase, MI = myocardial infarction, miR = microRNA, MPP^+^ = 1-methyl-4-phenylpyridinium, MS = multiple sclerosis, MSC = mesenchymal stem cell, mTOR = mechanistic target of rapamycin, NEDD = neuronally expressed developmentally down-regulated, NF-κB = nuclear factor-kappa B, NFE2L2 = nuclear factor erythroid-2 related factor-2, NSC = neural stem cell, OA = osteoarthritis, oe = overexpressing, OE = olfactory ecto, PD = Parkinson’s disease, PD-L1 = programmed death ligand 1, PDLSC = periodontal ligament stem cell, PI3K = phosphoinositide 3-kinase, POI = premature ovarian insufficiency, PTEN = phosphatase & tensin homolog, RA = rheumatoid arthritis, RGD = arginine-glycine-aspartic acid, SC = stem cell, SCI = spinal cord injury, SHED = stem cells from human exfoliated deciduous teeth, SNI = sciatic nerve injury, sphk = sphingosine kinase, STAT = signal transducers and activators of transcription, TAZ = transcriptional co-activator with PDZ-binding motif, Tβ = thymosin, TBI = traumatic brain injury, tfh = T follicular helper, TGF-β = transforming growth factor-β, TLR = toll-like receptor, TNF = tumor necrosis factor, TRAF = TNF receptor-associated factor, TSG = TNF stimulated gene, UC = umbilical cord, USC = urine stem cell, VEGF = vascular endothelial growth factor, YAP = yes-associated protein, YY = Yin Yang)

### The PTEN/PI3K/Akt/mTOR pathway in orthopedic diseases

In terms of fracture treatment using SC-Exo, Liang et al. preconditioned parental MSCs with low doses of dimethyloxaloylglycine (DMOG), a small angiogenic molecule, to prepare the exosomes for an enhanced bone regeneration and angiogenesis in a critical-sized calvarial defect model [29]. In comparison to systemic administration, local delivery of SC-Exo using single or composite biomaterials could provide comparable results. Liu et al. revealed that exosomal miR-130a delivered by lithium-incorporated bioglass scaffold could support pro-angiogenic activity [30], whereas Hu et al. found that composite scaffold containing bioglass and GelMA/nanoclay hydrogel (HG) could transfer exosomal miR-23a-3p from umbilical cord (UC) MSCs for vascularized bone regeneration [31].

In terms of osteoarthritis (OA) treatment, Wu et al. discovered that miR100-5p-abundant exosomes derived from infrapatellar fat pad MSC could protect articular cartilage from damage and ameliorate gait abnormality in mice [32]. Meanwhile, Shen et al. loaded miR-205-5p from hypoxia-preconditioned BM MSCs in an injectable silk fibroin (SF) HG to improve cartilage regeneration in diarthrodial joints [33].

In terms of treatment of intervertebral disc (IVD) degeneration (IDD), Cheng et al. showed that intradiscal injection of BM MSC-derived exosomes could inhibit nucleus pulposus cell (NPC) apoptosis and alleviate IDD via exosomal miR-21 [34]. Luo et al. achieved similar therapeutic results by locally delivering cartilage endplate stem cells-derived exosomes from an injectable costal cartilage extracellular matrix (ECM) HG [35].

### The PTEN/PI3K/Akt/mTOR pathway in neurosurgical diseases

In terms of treatment of spinal cord injury (SCI), some groups targeted anti-inflammation and immunomodulation whereas others targeted angiogenesis and blood-spinal cord barrier (BSCB). Liu et al. discovered that not only hypoxia preconditioning could increase exosome yield from bone marrow MSCs, exosomal miR-216a-5p could also repair traumatic SCI by shifting microglial M1/M2 polarization [36]. Chen et al. loaded neural stem cell (NSC)-derived exosomes with FTY720, a microvascular regulator and immune modulator, to protect the barrier function of spinal cord microvascular endothelial cells (SCMECs), thereby improving hindlimb function in rats after SCI [37]. When delivered locally, SC-Exo demonstrated comparable effects in SCI repair. The work from Cao et al. targeted angiogenesis. It was found that urine stem cells-derived exosomes and exosomal ANGPTL3 locally delivered by an injectable HG could promote spinal cord functional recovery by enhancing angiogenesis [38]. In comparison, the work from Fan et al. used an electroconductive HG for the local delivery of BM MSC-derived exosomes and targeted neuroinflammation [39]. It was demonstrated that this strategy could synergistically promotes tissue repair after SCI via immunoregulation and enhancement of myelinated axon growth.

In terms of treatment of ischemic stroke, Yoon et al. enhanced the anti-inflammatory effect of SC-Exo therapy by modifying the exosomes. They increased the yield of NSC-derived exosomes by creating tumor susceptibility gene (TSG)101-overexpressing human NSCs [40]. The engineered exosomes reduced LDH level and proinflammatory cytokines in vitro while lowering infarction volume and increasing production of neurotrophic factors in vivo.

### The PTEN/PI3K/Akt/mTOR pathway in plastic surgical diseases

Cutaneous wound healing is the most common indication in plastic surgery for SC-Exo therapy. The general phases of wound healing include hemostasis, inflammation, angiogenesis, proliferation and remodeling [41]. All reported work using SC-Exo therapy to enhance wound healing used MSC-derived exosomes.

On the one hand, this remedy could be conducted via systemic injection. Targeting the angiogenic stage, Ding et al. pre-conditioned BM MSC-derived exosomes with deferoxamine, a hypoxia-mimetic that simulates the oxygen deprivation effects [42]. The exosomal miR-126 accelerated cutaneous wound healing by improving angiogenesis in rats with streptozotocin-induced diabetic wound. Targeting the proliferative stage, Zhang et al. used adipose MSC-derived exosomes to promote fibroblast proliferation and migration at the cellular level, thereby optimizing collage deposition in the wound at tissue level [43].

On the other hand, this remedy could be performed via local delivery, especially targeting the inflammation stage of wound healing. For example, Geng et al. fabricated a multifunctional antibacterial and self-healing HG for local delivery of BM MSC-derived exosomes, which could inhibit inflammation by M2 macrophage polarization [44]. Jiang et al. developed a smart HG based on an enzyme responsive MMP/PEG [45]. The HG-assisted local delivery of adipose MSC-derived exosomes could enhance diabetic wound healing in mice by optimizing cellular behaviors and ameliorating oxidative stress.

### The PTEN/PI3K/Akt/mTOR pathway in other diseases

In terms of treatment of liver fibrosis, Ma et al. revealed that circDIDO1-overexpressing MSC-derived exosomes could inhibit hepatic stellate cell activation by exosomal miR-141-3p in human liver fibrosis [46]. Liver fibrosis is a hepatic disease commonly managed by general surgeons. It happens when the liver sustains a chronic insult, which may progress into liver cirrhosis, liver failure, hepatocellular carcinoma, and eventually fatality [47].

In terms of treatment of ischemic heart disease (IHD), Wen et al. focused on the death of cardiomyocytes because prolonged oxygen deprivation to the myocardium might lead to cellular death. They found that MSC-derived exosomes could alleviate cardiomyocyte apoptosis in hypoxic conditions through exosomal miR-144 [48]. IHD, along with other closely related cardiothoracic diseases, such as atherosclerosis, myocardial infarction (MI), myocardial ischemia reperfusion injury, and even heart failure, might also become indications for SC-Exo therapy.

In terms of treatment of microtia, a congenital ear abnormality and a challenging deformity for the otorhinolaryngology surgeons, Chen and co-workers focused on the preclinical large-scale production and functional modulation of adipose MSC-derived exosomes using porous GelMA HG [49]. They first genetically engineered adipose MSCs from microtia patients cultured on dual functional HG to produce miR-23a-3p-rich exosomes. Then, the exosomal miR-23a-3p were incorporated into microtia chondrocytes to improve cell survival and proliferation, inhibit cell apoptosis, and promote tissue-engineered ear cartilage regeneration.

### Typical mechanism of action of SC-Exo therapy in regulating the PTEN/Akt pathway

In an in vitro study, MSC-derived exosomes could ameliorate cardiomyocyte apoptosis in hypoxic conditions through miR-144 by targeting the PTEN/Akt pathway [48]. Firstly, exosomes were easily internalized by H9C2 cells, and exosome-mediated protection from apoptosis was associated with increased levels of phosphorylated Akt. Secondly, miR-144 was discovered to be highly enriched in MSC-derived exosomes. Transfection of cells with a miR-144 antagonist could not only diminish exosome-mediated protection from apoptosis but also upregulate PTEN and downregulate p-Akt expression in hypoxic conditions. Lastly, PTEN was proven to be a target of miR-144 using luciferase reporter assay, and cells treated with a PTEN-specific inhibitor could increase p-Akt expression and reduce H9C2 cell apoptosis. These mechanistic explorations demonstrated that MSC-derived exosomes represent a promising vehicle to facilitate delivery of miRNA therapies to functionally improve ischemic heart diseases such as myocardial infarction.

## The NF-κB pathway regulated by SC-Exo therapy

**Fig. 4:**
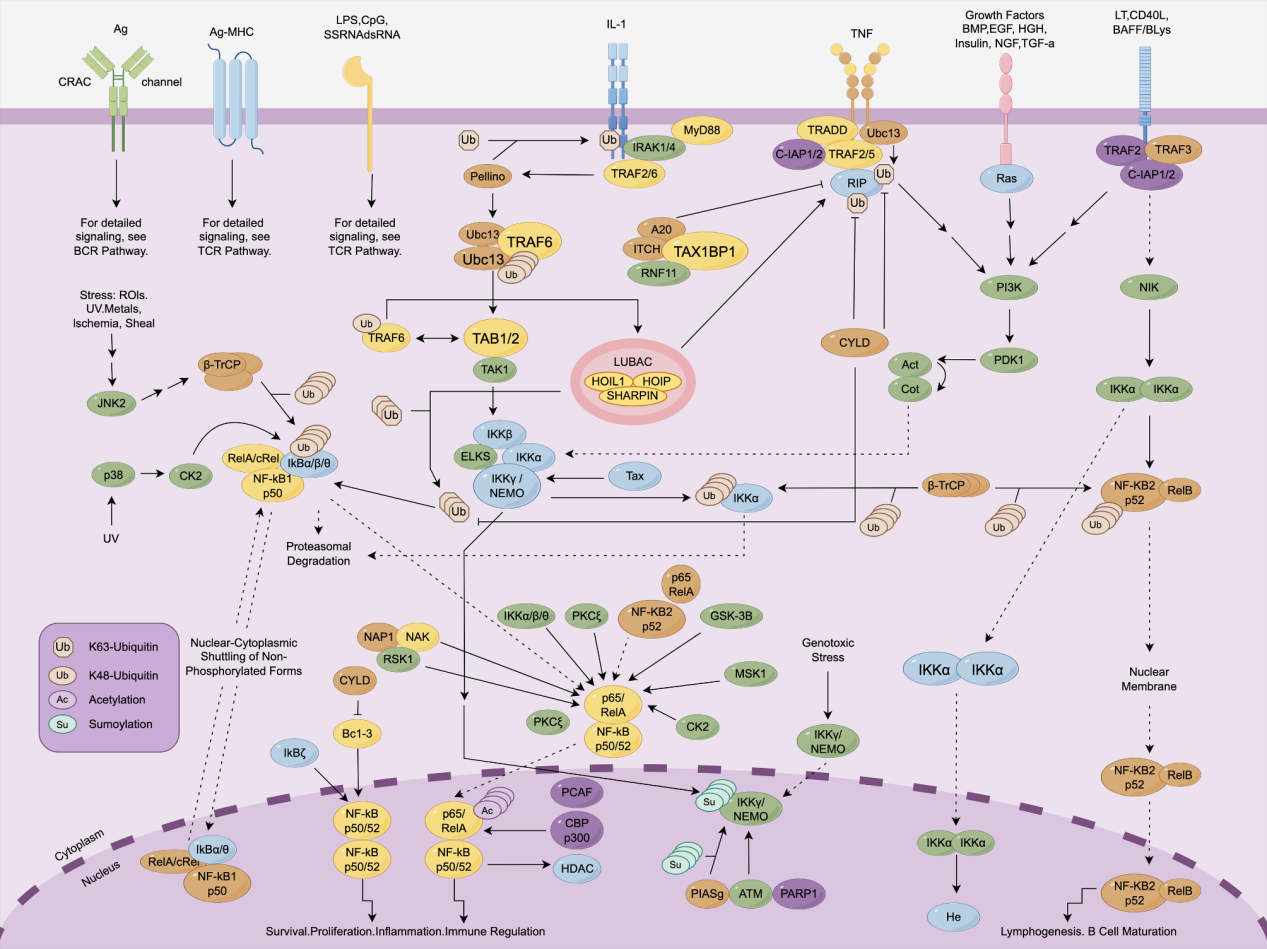
Schematic diagram of the NF-κB signaling pathway. Figure generated using FigDraw online platform (www.figdraw.com) with an export ID of WWYUIe8747

Since its discovery just over 30 years ago, nuclear factor kappa B (NF-κB), a family of rapidly inducible transcription factors, has been found to play crucial roles in both physiological and pathological processes [50]. The NF-κB family contains five members, i.e., p65 (RelA), RelB, c-Rel, p105/p50, and p100/p52, with the RelA-p50 heterodimers and RelB-p52 heterodimer accountable for the canonical/classical and non-canonical/alternative NF-κB pathways, respectively [51]. Various signals and stimuli can activate the canonical NF-κB pathway, such as TNF receptor (TNFR) superfamily members, pattern-recognition receptors (PRRs), T-cell receptor (TCR), B-cell receptor (BCR), and ligands of multiple cytokine receptors (Fig. 4). The primary event in NF-κB activation is the inducible phosphorylation of IκB (inhibitor of NF-κB) proteins by IKKs (IκB kinases) [52]. Subsequently, the ubiquitination-dependent degradation by proteasome releases κB transcription factor from cytoplasmic inhibition to translocate to nucleus and activate the target genes.

The NF-κB pathway serves as a pivotal mediator of various diseases (e.g., autoimmune, inflammatory, and cancer) and plays a key role in many biological processes (e.g., autophagy, cellular senescence and metabolism, tissue regeneration and repair) [53]. In particular, the canonical NF-κB pathway has been historically deemed a prototypical pro-inflammatory signaling cascade, mainly because of NF-κB’s contribution to the expression of various pro-inflammatory genes and participation in inflammasome regulation [54]. The activation of NF-κB is associated with multiple chronic inflammatory conditions such as rheumatoid arthritis (RA), multiple sclerosis (MS), atherosclerosis, chronic obstructive pulmonary disease (COPD), asthma, inflammatory bowel disease (IBD), and ulcerative colitis (UC) [55].

### The NF-κB pathway in orthopedic diseases

In terms of treatment of tendon injury, some research groups targeted individual components of the muscle-tendon-bone complex. For example, Jin et al. showed that PDA-modified GelMA microspheres could encapsulate exosomes derived from young stem cells of human exfoliated deciduous teeth for local delivery [56]. This strategy could ameliorate senescent phenotypes in aged tendon stem cells and suppress ectopic bone formation in naturally aging mice, thereby restoring endogenous tendon regeneration.

In terms of RA treatment, Tian et al. demonstrated that gingival MSC-derived exosomes could be immunosuppressive in preventing collagen-induced arthritis [57]. Gingival MSC-derived exosomes exhibited similar or stronger effects than parental stem cell in downregulating IL-17 A and upregulating IL-10, as well as reducing incidences and severity of and bone erosion. This was achieved through inhibiting the IL-17RA-Act1-TRAF6NF-κB signaling pathway.

In terms of treatment of growth plate injury, a common cause for delayed bone bridge formation and limb length discrepancy in pediatric population, Guan et al. loaded BM MSC-derived exosomes in an ECM-mimicking HG composed of GelMA and aldehyde-functionalized CS for locally delivery [58]. This system could enhance the synthesis of ECM owing to CS doping and suppress the inflammation of chondrocytes, thereby stimulating the repair of growth plate injury.

### The NF-κB pathway in neurosurgical diseases

Neurosurgical diseases have been popular indications for SC-Exo therapy targeting the NF-κB pathway. In terms of treatment of traumatic brain injury (TBI), Chen et al. systemically injected adipose MSC-derived exosomes [59]. They discovered that SC-Exo therapy could promote functional recovery, inhibit neuroinflammation, reduce neuronal apoptosis, and improve neurogenesis through suppressing microglia/macrophage activation. In comparison, Hajinejad et al. designed a 3D nano-scaffold expressing a bio-motif of stromal cell-derived factor (SDF)-1α for the local delivery of NSC-derived exosomes [60]. This intelligent platform reduced oxidative stress and reactive gliosis-related neuroinflammation, and elevated neurogenesis through a bio-bridge mechanism.

In terms of SCI treatment, both systemic and local delivery have been trialed. Ma et al. used insulin-like growth factor 1 (IGF-1) to stimulate NSC-derived exosomes for an enhanced neuroprotective and anti-neuroinflammatory effect [61]. This SC-Exo therapy was found to attenuate neuronal apoptosis while improving functional recovery after SCI through upregulation of miR-219a-2-3p and downregulation of YY1. In comparison, Fan et al. developed an electroconductive GelMA/PPy HG for the local delivery of BM MSC-derived exosomes [39]. This solution could regulate microglial M2 polarization and synergistically improve neuronal and oligodendrocyte differentiation of NSCs while suppressing astrocyte differentiation. Compared to SCI, peripheral nerve injury such as sciatic nerve injury (SNI) is more common. Yang et al. used self-curling electroconductive TA/PPy HG for local delivery of BM MSC-derived exosomes [62]. This laminar HG dressing excelled by automatically wrapping around the damaged nerve fibers to form a size-matched tube-like structure, thereby avoiding the cumbersome implantation process. In addition, it could simultaneously achieve functional recovery and pain relief.

In terms of treatment for other neurosurgical diseases, systemic administration of MSC-derived exosomes has been successfully used in preclinical studies for ischemic stroke and MS. In one study, human UC MSC-derived exosomal miR-146a-5p was proven to reduce microglia-mediated neuroinflammation while attenuating behavioral deficits through the IRAK1/TRAF6 coupling after ischemic stroke [63]. In the other study, BM MSC-derived exosomes were found to increase remyelination and reduce neuroinflammation in the demyelinating central nervous system (CNS) of animals using both experimental autoimmune encephalomyelitis and cuprizone diet models [64].

### The NF-κB pathway in other diseases

In terms of treatment of cutaneous wound healing, both original and modified exosomes have been used for topical SC-Exo therapy. Targeting the proliferative stage of wound healing, Liu et al. showed that adipose MSC-derived exosomes released from β-chitin nanofiber HG could accelerate wound closure through complement factor D and its downstream Aldolase A and Actn2 proteins in rats [65]. Targeting multiple stages of wound healing, Li et al. first engineered placental MSC-derived exosomes with miR-146a, then locally delivered them using a silk fibroin (SF) patch [66]. This strategy could improve diabetic wound healing by inhibiting inflammation and enhancing collagen deposition and neovascularization in the neo-epithelium.

In terms of treatment of chronic endometritis, Zhao et al. pre-conditioned BM MSC-derived exosomes with interleukin (IL)-1b for a more potent anti-inflammatory effect [67]. Local delivery of these activated exosomes using an injectable polypeptide HG scaffold could upregulate anti-inflammatory factors and downregulate pro-inflammatory factors in vitro while supporting endometrial regeneration in vivo.

### Typical mechanism of action of SC-Exo therapy in regulating the NF-κB pathway

In an in vivo study, modification of specific EPC-derived exosomal cargo could rescue their reparative activity through modulating the NF-κB pathway [68]. Firstly, in a mouse model of MI, wild-type EPC-derived exosome treatment was found to improve left ventricle cardiac function, suppress cardiomyocyte apoptosis, reduce MI scar size, and enhance post-MI angiogenesis, whereas IL-10 knockout EPC-derived exosome treatment demonstrated opposite effects. Secondly, mass spectrometry discovered that wild-type EPC-derived exosome and IL-10 knockout EPC-derived exosome manifest different protein expression pattern. Specifically, integrin-linked kinase (ILK) was highly enriched in both IL-10 knockout EPC-derived exosome and TNF-α inflamed mouse cardiac EPC-derived exosome. Lastly, ILK-enriched exosomes could activate the NF-κB pathway and NF-κB-dependent gene transcription in recipient endothelial cells, and this effect was counteracted via ILK knockdown in exosomes. These mechanistic explorations provided new insights of how inflammation might change stem cell-exosome-mediated cardiac repair.

## The TGF-β pathway regulated by SC-Exo therapy

The transforming growth factor-beta (TGF-β) superfamily of secreted, homodimeric and heterodimeric proteins exhibit pleiotropic effects on many cell types, and are implicated in diverse aspects of cellular and tissue physiology [69]. The effects of TGF-β signal transduction pathway are cellular context-dependent, on cell type, growth phase, differentiation status, and epigenetic state [70]. The TGF-β signaling could induce cytostasis in some cells, but also determine cellular behaviors such as proliferation, apoptosis, autophagy, senescence, and dormancy in others [71]. On the other hand, dysregulation of TGF-β pathway leads to various pathologies, such as inflammatory progression and immune overreaction, fibrotic diseases and improper wound healing, and cancer migration and invasion [72]. Thus, the TGF-β signaling pathway has become a popular target for ongoing clinical trials and drug development [73].

**Fig. 5:**
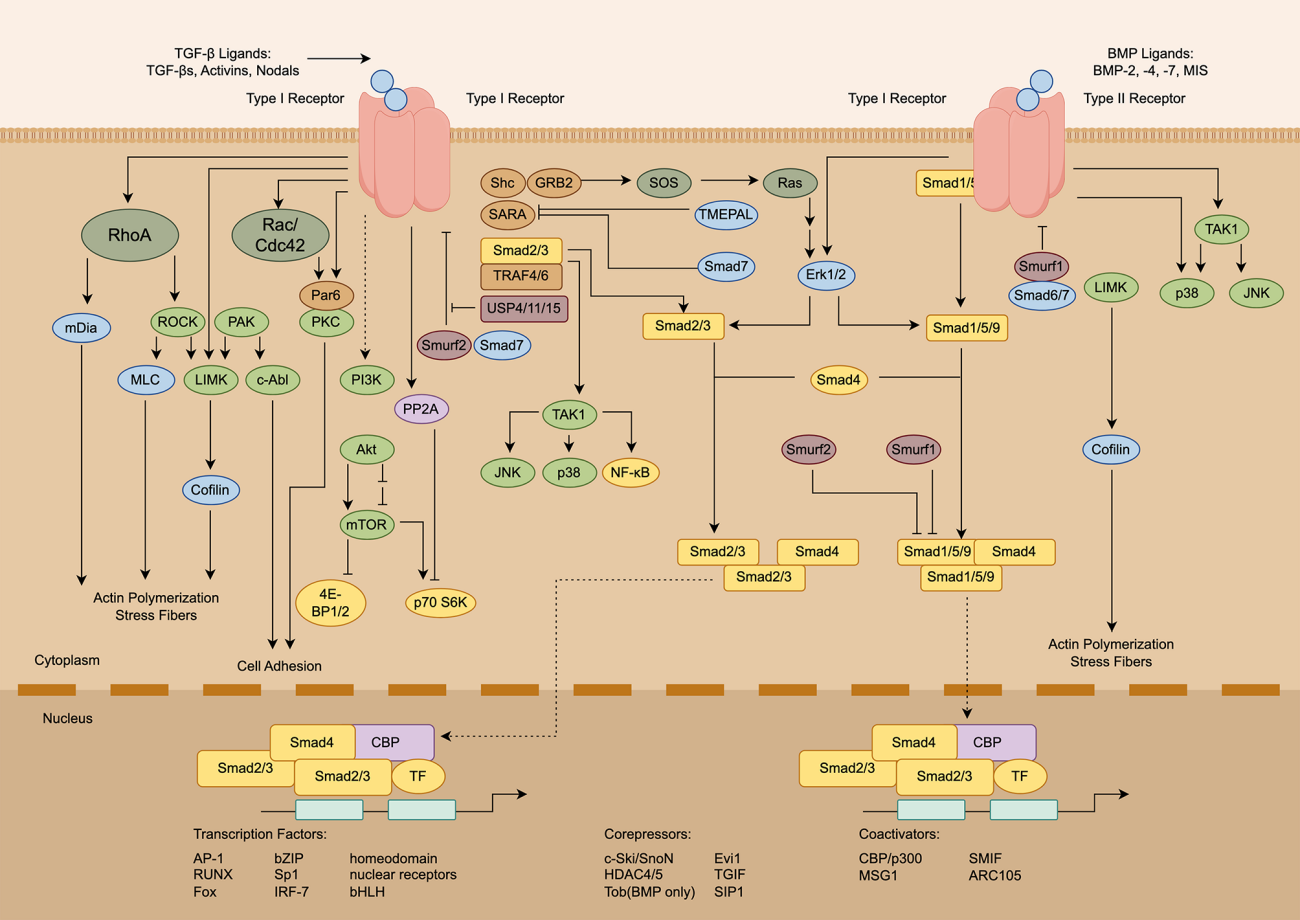
Schematic diagram of the TGF-β signaling pathway. Figure generated using FigDraw online platform (www.figdraw.com) with an export ID of UAATId47b7

The signaling mechanisms of the TGF-β family are controlled at multiple levels. At the extracellular level, the signaling is initiated by interaction with integrins, proteolytic cleavage, and temperature and pH changes (Fig. 5). At the cytoplasmic level, TGF-β proteins link with serine/threonine receptor kinases, phosphorylate downstream mediators (e.g., Smad2 and Smad3), and transduce signaling to ubiquitin ligases and intracellular protein kinases [74]. At the nucleus level, activated Smads mediate regulatory signals that control the expression of target genes, and eventually nuclear or cytoplasmic protein regulation. More importantly, the TGF-β pathway heavily interacts with other signaling cascades, such as the PI3K, JNK, p38, NF-κB, ERK, and Wnt/β-catenin, EGFR, and BMP-7 signal transduction pathways [75].

### The TGF-β pathway in orthopedic diseases

In terms of fracture treatment, Liu et al. used a hierarchical mesoporous bioactive glass scaffold instead of previously discussed hydrogel, for local delivery of exosomes [76]. Using a rat cranial defect model, the authors proved that the loading of BM MSC-derived exosomes could enhance the bone forming capacity of the scaffold and induce rapid initiation of bone regeneration via the Bmpr2/Acvr2b competitive receptor-activated Smad signaling.

In terms of treatment of tendon injury, Liu et al. used GelMA HG to locally release adipose MSC-derived exosomes [77]. This system could stimulate the proliferation, migration, and tenogenic differentiation of tendon stem cells while suppressing inflammatory reaction and improving tendon healing in rats. These comprehensive effects were achieved via activation of both SMAD1/5/9 and SMAD2/3 pathways.

### The TGF-β pathway in neurosurgical diseases

In terms of SCI treatment, Nakazaki et al. demonstrated that intravenous infusion of MSC-derived exosomes could stabilize the BSCB and improve locomotor recovery in experimental models of SCI [78]. Although IV administered MSCs do not travel to the injury site, IV delivered exosomes do and were taken up mainly by a subset of M2 macrophages, which was evidenced by DiR-labelling. In addition, fractionated dosing of SC-Exos over 3 days provided comparable therapeutic effect with a single MSC injection on multiple parameters.

### The TGF-β pathway in plastic surgical diseases

The TGF-β pathway plays a central role in both wound healing and scar formation [79]. All reported work using SC-Exo therapy to enhance wound healing via TGF-β pathway used UC MSC-derived exosomes. Hu et al. and Fang et al. both used systemic administration of exosomes targeting the last phase of wound repair (i.e., remodeling stage) during which abnormal wound healing such as hypertrophic scars might happen. On a higher level than TGF-β, cellular and tissue abnormalities such as excessive inflammation and angiogenesis, dysregulated matrix metalloproteinases, and delayed apoptosis of fibrotic myofibroblasts all contribute to the pathogenesis of hypertrophic scars [80]. Both teams discovered that UC MSC-derived exosomes could attenuate dermal fibroblast-myofibroblast transition, thereby diminishing excessive scar formation. The former group found that this effect was achieved via inhibition of the TGF-β1/Smad 2/3 signaling pathway as evidenced by significantly reduced levels of Collagen I, Collagen III, α-SMA, and Smad2/3, and Smad2/3 phosphorylation in fibroblasts after SC-Exo therapy [81]. The latter group discovered that exosomal microRNAs (e.g., miR-21, miR-23a, miR-125b, and miR-145) played key roles in inhibiting myofibroblast formation by suppressing the TGF-β2/Smad2 pathway using high-throughput RNA sequencing and functional analysis [82]. On the other hand, some team applied biomaterials for local delivery of SC-Exo. For example, Yang et al. used a thermosensitive pluronic F127 HG for local delivery of UC MSC-derived exosomes [83]. This combination upregulated CD31, Ki67, VEGF and TGF-β2 expression, thereby improving chronic wound healing and complete skin regeneration in a streptozotocin-induced diabetic rat model.

In terms of scleroderma treatment, Li et al. systemically injected UC MSC-derived exosomes in a murine model of bleomycin-induced scleroderma [84]. The SC-Exo therapy ameliorated dermal fibrosis by inhibiting both myofibroblast activation and collagen deposition via downregulation of the TGF-β/Smad signaling cascade.

## The HIF-1α pathway regulated by SC-Exo therapy

**Fig. 6:**
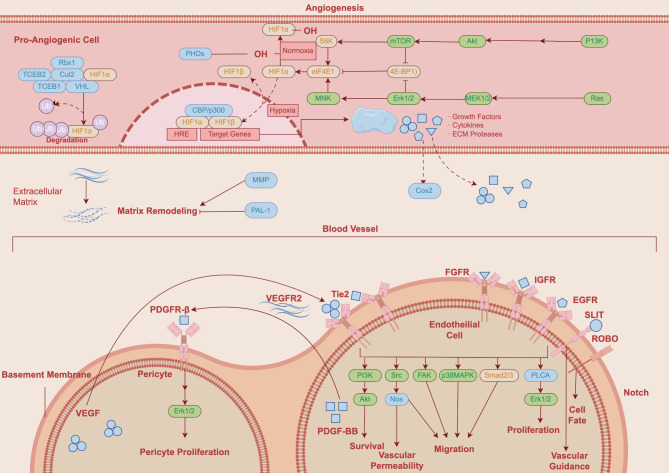
Schematic diagram of the HIF-1α signaling pathway. Figure generated using FigDraw online platform (www.figdraw.com) with an export ID of OPIPY19917

Cellular hypoxia occurs when oxygen demand exceeds supply, thereby leading to a state of oxygen depletion and energetic crisis. It is a common feature in immunity, inflammation, and tumor microenvironment [85]. With an attempt to protect against hypoxic threat, cells could initiate oxygen-sensitive adaptive transcriptional responses, such as the hypoxia-inducible factor (HIF) pathway, thereby promoting adaptation to hypoxia by upregulating genes responsible for angiogenesis, erythropoiesis, and glycolysis [86]. Under normoxia, the proline residues of HIF-1α subunits are hydroxylated by the oxygen-sensing prolyl hydroxylase domains (PHDs) (Fig. 6). The transcriptional activity of HIF-1α subunits is controlled by the factors inhibiting HIFs (FIHs). Under hypoxia, the activity of both PHDs and FIHs are inhibited, while HIF-1α subunits translocate into the nucleus to combine with HIF-1β (Fig. 6). Then, the heterodimeric HIF-1α/HIF-1β transcription factor complex travels to the hypoxia-responsive elements (HREs) of its target genes, leading to subsequent transcriptional modulation [87].

The biological implication of the HIF-1α pathway is reflected at multiple levels. Firstly, targeting HIF-1α pathway has shown tremendous potential in the treatment of human diseases at various organs including heart (e.g., ischemic heart disease, congestive heart failure), lung (e.g., acute lung injury, pulmonary fibrosis), and liver (e.g., acute liver failure) [88]. Secondly, HIF-1α pathway participates in various biological processes, such as angiogenesis, metabolic homeostasis, immune response, microbial infection, and tumor progression [89]. Lastly, HIFs act as a master switch in angiogenesis and vascular remodeling (Fig. 6) [90, 91]. In addition, the HIF-1α signaling has extensive crosstalk and synergism with other pathways such as the NF-κB, ERK, Wnt/β-catenin, Notch, and PTEN/PI3K/Akt/mTOR pathways.

### The HIF-1α pathway in orthopedic diseases

In terms of fracture healing, umbilical cord MSC-derived exosomes showed comparable results to bone marrow MSC-derived ones. Moreover, exosomes produced under hypoxia conveyed stronger therapeutic effects than those under normoxia [92]. In detail, hypoxia preconditioning promoted exosomal miR-126 production through activating SPRED1/Ras/Erk signaling and HIF-1α pathway. Furthermore, multiple studies have demonstrated that hypoxia pre-treatment represents a potent and promising optimization of the therapeutic effects of SC-Exo for bone fracture treatment [93]. On the other hand, few projects used HG, especially those from modified natural polymers, as the biomaterial for local delivery of SC-Exo for fracture healing. For example, Zhang et al. used commercial hyaluronic acid (HA)-based semi-synthetic HG supplemented by heparin sulfate, aka HyStem™ HP [94]. They showed that UC MSC-derived exosomes could promote fracture healing through HIF-1α mediated elevation of angiogenesis in a rat model of stabilized fracture.

### The HIF-1α pathway in neurosurgical diseases

In terms of SCI treatment, rather than targeting neuroprotection, neuroregeneration, anti-inflammation and immunomodulation, some groups focused on the angiogenesis aspect of SCI healing. Mu et al. fabricated an adhesive peptide-modified HA HG for local delivery of hypoxia-preconditioned UC MSC-derived exosomes [95]. In vitro, this system protected human vascular cells from oxygen-glucose deprivation (OGD) while increasing their migration and tube formation. In vivo, the released exosomal HIF-1α promoted the production of VEGF and angiogenesis and locomotor function in rats.

### The HIF-1α pathway in plastic surgical diseases

Cutaneous wound healing is the most common indication for SC-Exo therapy in plastic surgery. On the one hand, systemic administration of SC-Exo targeted the proliferative stage of wound healing. Zhang et al. discovered that adipose MSC-derived exosomes could promote wound healing through accelerated keratinocyte migration and proliferation by activating the Akt/HIF-1α signaling cascade [96]. On the other hand, regional delivery of SC-Exo targeted the angiogenic stage of wound healing using GelMA HG as a conveyer. Hu et al. showed that circ-Snhg11-modified adipose MSC-derived exosomes could increase the migratory, proliferative and blood vessel regeneration potential of vascular endothelial cells through activation of the miR-144-3p/NFE2L2/HIF-1α signaling cascade [97]. Wang et al. reported that VH298-loaded, epidermal stem cell-derived exosomes could facilitate diabetic wound healing via the HIF-1α/VEGFA signaling cascade [98].

### The HIF-1α pathway in other diseases

In terms of treatment of MI, Chen et al. developed artificial stem cells that could sustainably release Thymosin β4 (Tβ4)-exosomes by encapsulating SC-Exo within microspheres using microfluidics technology, thereby mimicking the paracrine activity of stem cells [99]. The exosomal miR-17-5p could ameliorate coronary collateralization in the periphery of infarcted area and cardiac functions, with a therapeutic effect superior to that of direct systemic injection of the exosomes.

## The Wnt pathway regulated by SC-Exo therapy

**Fig. 7:**
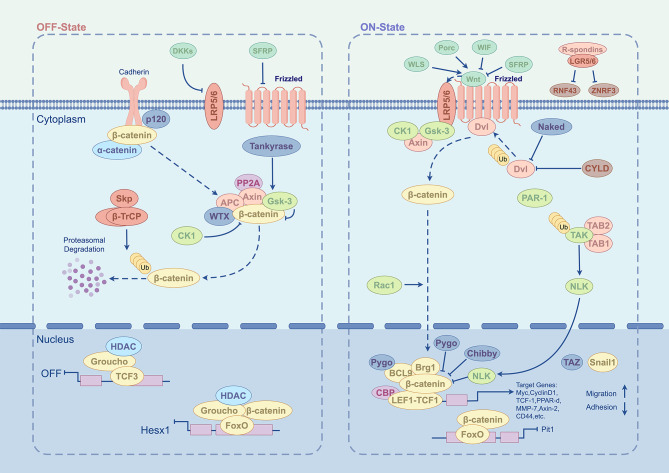
Schematic diagram of the Wnt signaling pathway. Figure generated using FigDraw online platform (www.figdraw.com) with an export ID of YUURP4ba86

Wnt glycoproteins are a family of growth factors containing 19 members and acting as short or long range signaling molecules [100]. The canonical Wnt pathway functions by regulating the transcriptional co-activator β-catenin, thereby controlling embryonic development and adult homeostasis [101]. The Wnt/β-catenin pathway is composed of four main groups of components: the extracellular signals, cell membrane units, cytoplasmic segments, and nuclear components [102]. The extracellular signals are typically activated by Wnt proteins including Wnt3a, Wnt1, and Wnt 5a (Fig. 7). The membrane units comprise the Wnt receptors Frizzled and LRP5/6. The cytoplasmic segments transmit signals through β-catenin, DVL, GSK-3β, AXIN, APC and CK1. The nuclear components mainly include TCF/LEF family members and β-catenin downstream target genes involved in cell survival, proliferation, migration and differentiation.

Dysregulation of Wnt/β-catenin pathway is associated with various developmental disorders, neurodegenerative diseases, chronic bone conditions, cardiovascular problems, and cancers [103]. In addition, Wnt/β-catenin signaling plays crucial roles in stem cells by maintaining their pluripotency, initiating their differentiation, and guiding their lineage commitment [104]. For example, driving Wnt/β-catenin signaling facilitates the homeostatic self-renewal and damage repair of Wnt-dependent stem cells, thereby promoting long-term organoid growth [105, 106]. It appeared that manipulation of Wnt pathway by SC-Exo therapy were achieved mostly through systemic administration of exosomes.

### The Wnt pathway in orthopedic diseases

MicroRNA (miRNA) is a small non-coding regulatory RNA that regulate gene expression at the post-transcriptional level. Exosomal miRNA has demonstrated diagnostic and therapeutic potential in various diseases [107, 108]. In terms of fracture treatment, Yu et al. revealed that exosomal miR-136-5p from bone marrow MSCs could stimulate osteoblast proliferation and differentiation while promoting fracture healing by inhibiting the downstream target gene, low-density lipoprotein receptor-related protein 4 (LRP4) of the Wnt/β-catenin pathway [109]. In terms of OA treatment, Mao’s and Tao’s teams both demonstrated similar results. The former proved that BM MSC-derived exosomes could enhance chondrogenesis and suppress cartilage degradation in mice, during which the overexpressing exosomal miR-92a-3p acted as a Wnt inhibitor and disease-modifying OA drug (DMOAD) [110]. The latter suggested that exosomes derived from miR-140-5p-overexpressing synovial MSCs could improve cartilage regeneration and prevent knee OA in rats through manipulating Wnt5a and Wnt5b [111].

### The Wnt pathway in neurosurgical diseases

In terms of treatment of ischemic stroke, Wei et al. showed that Zeb2/Axin2-enriched bone marrow MSC-derived exosomes could promote post-stroke functional recovery by enhancing neurogenesis, neural plasticity, and spatial memory [112]. This was most likely achieved through downregulating the SOX10, endothelin-3/EDNRB, and Wnt/β-catenin pathways.

### The Wnt pathway in plastic surgical diseases

In terms of treatment of cutaneous wound, He et al. demonstrated that adipose MSC-derived exosomes containing MALAT1 could mediate H_2_O_2_-induced wound healing by targeting miR-124 in human epidermal keratinocytes and fibroblasts [113]. In terms of alopecia treatment, Cao et al. showed that NSC-derived exosomes could stimulate hair follicle growth via miR-100 in depilation-induced mice [114].

## The MAPK pathway regulated by SC-Exo therapy

**Fig. 8:**
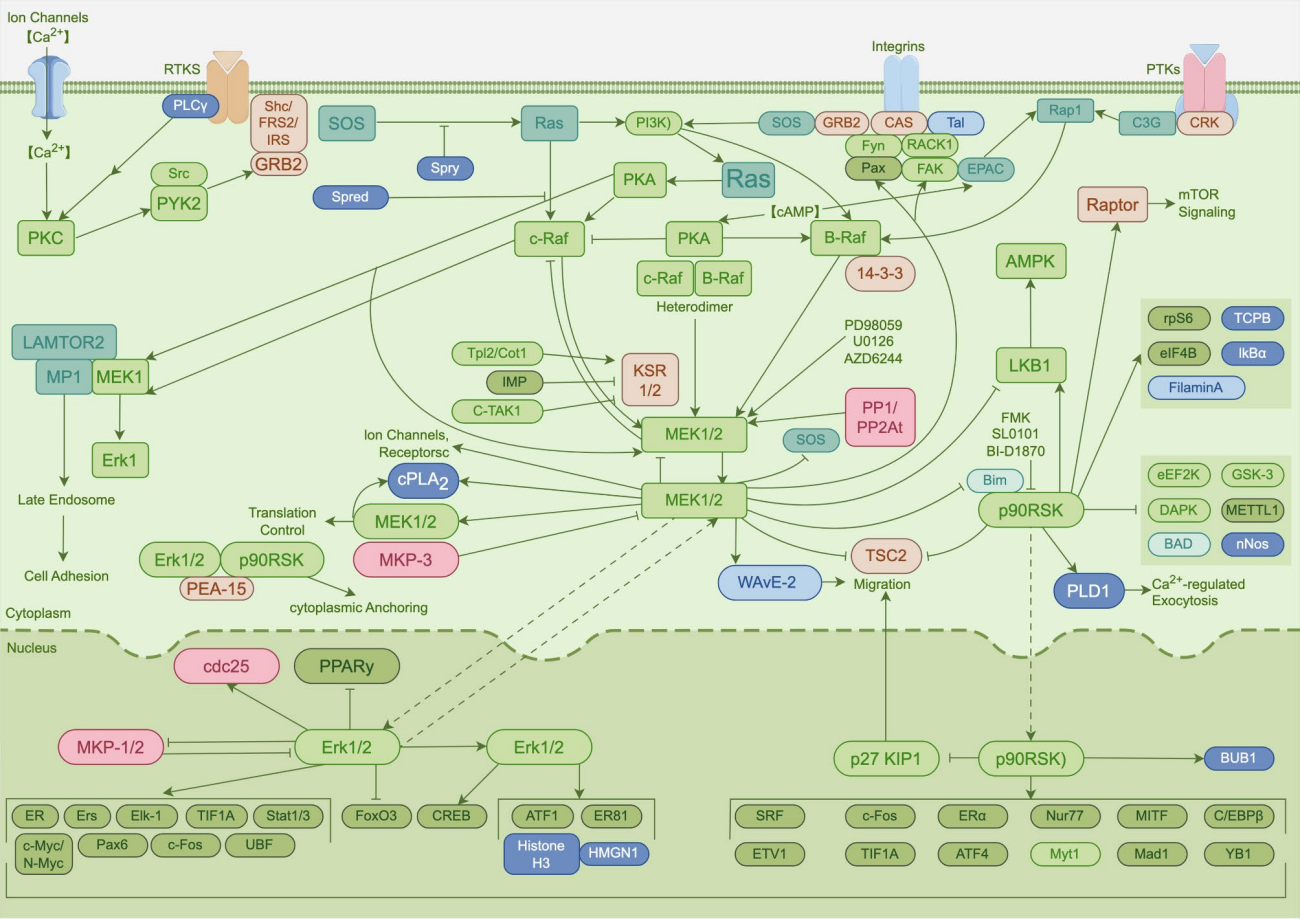
Schematic diagram of the MAPK signaling pathway. Figure generated using FigDraw online platform (www.figdraw.com) with an export ID of RYYROb03b9

The mitogen-activated protein kinase (MAPK) cascades play a crucial role in many cellular processes including cell proliferation, differentiation, apoptosis, metabolism, motility, stress response and inflammation [115]. At least three MAPK families have been recognized and characterized, i.e., classical MAPK or extracellular signal-regulated kinase (ERK), Jun kinase (JNK) and p38 kinase. Each of these signaling cascades comprises at least three tiers of enzymes that are sequentially activated [116]. In detail, activation of the conventional MAPK pathway occurs at cell membrane where various protein or receptor kinases phosphorylate MAPKKKs. They then directly activate MAPKKs which phosphorylate MAPKs (Fig. 8). The sequentially activated kinases interact with numerous downstream substrates and ultimately regulate transcription factors that push context-specific gene expression [117].

As one of the most ancient signal transduction pathways, MAPK cascades exhibit a central role in innate immune responses, ranging from activation of anti-inflammatory feedback loop to induction of pro-inflammatory mediators [118]. For example, p38α could activate MAPK-activated protein kinase 2 (MK2) which in turn upregulate TNF production, whereas ERK1/2 could activate mitogen- & stress-activated kinases (MSKs) which in turn upregulate IL-10 and IL-1RA production, thereby providing evidence for the development of MAPK inhibitors for anti-inflammatory therapy [119]. Most SC-Exo therapy targeting MAPK pathway were given systemically and to neurosurgical diseases.

### The MAPK pathway in neurosurgical diseases

In terms of TBI treatment, Chen et al. discovered that adipose MSC-derived exosomes injected through the venous system could specifically enter microglia/macrophages and suppress their activation [59]. This in turn inhibited neuroinflammation and boosted functional recovery after TBI via inhibition of both NF-κB and MAPK pathways. In terms of SCI treatment, Zhou et al. reported that placental MSC-derived exosomes could enhance endogenous NSC proliferation through MEK/ERK/CREB cascade [120]. Ultimately, this effect not only promoted locomotor function but also relieved bladder dysfunction which is a frequent SCI complication that could further deteriorate patients’ quality of life. Compared to CNS injuries such as TBI and SCI, peripheral nerve injury such as sciatic nerve injury (SNI), is more common. In terms of SNI treatment, Yang et al. used a self-curling electroconductive HG for local delivery of SC-Exo [62]. The authors proved that BM MSC-derived exosomes could promote myelinated axonal regeneration and relieve inflammatory pain in diabetic rats through the NF-κB and MEK/ERK signaling cascades. Apart from traumatic nerve injury, ischemic stroke is also a neurosurgical indication for SC-Exo therapy. Tian et al. endowed NSC-derived exosomes with targeting ability to the ischemic lesion by attaching RGD peptide onto the exosomal membrane [121]. This ingenious system managed to inhibit the inflammatory response after cerebral ischemia.

### The MAPK pathway in urological diseases

Acute kidney injury (AKI) is characterized by reduced urine production and/or increased plasma creatinine. AKI is multifactorial in nature, with renal ischemia-reperfusion injury (IRI) as the most common etiology in inpatients [122]. Lim’s team demonstrated that exosomes from iPSC-derived MSCs could ameliorate renal damage following IRI via activation of the ERK1/2 signaling pathway [123]. The therapeutic effects were manifested by corrected serum creatinine level, tubular necrosis, inflammation and oxidative stress in mice with AKI.

## The JAK-STAT pathway regulated by SC-Exo therapy

Since its discovery 30 years ago, the Janus kinase/signal transducer & activator of transcription (JAK/STAT) signaling pathway has been deemed one of the central orchestraters in the cell function [124]. The JAK/STAT pathway is evolutionarily conserved, and comprises cytokines and growth factors (e.g., interferons, interleukins), JAKs (e.g., JAK1/2/3, TYK2), and STATs (STAT1/2/3/4/5a/5b/6) [125]. This pathway initiates by extracellular ligand binding upon activation of receptor-associated JAKs. Trans-phosphorylated JAKs then activate downstream STATs, followed by dimerization and translocation into the nucleus to regulate specific genes [126]. JAK/STAT-mediated downstream events are exemplified by immune regulation, tissue repair, apoptosis, and inflammation. Dysregulation of JAK/STAT pathway is implicated in multiple diseases including autoimmune disorders, allergic conditions, and cancers [127].

In terms of TBI treatment, Wen’s team suggested that bone marrow MSC-derived exosomes could suppress neuroinflammation and drive the transformation of microglia to the anti-inflammatory phenotype [128]. Meanwhile, the exosomal miR-181b could alleviate cell apoptosis in cortical tissue of mice with TBI via the IL-10/STAT3 pathway. In terms of treatment of Parkinson’s disease (PD), Huang et al. discovered that MSC exosomes-derived TSG6 with anti-inflammatory and anti-oxidative stress properties, could ease neurotoxicity in an in vitro PD model using SH-SY5Y and SK-N-SH cells [129]. In addition, the therapeutic effect was confirmed to be achieved through the STAT3/miR-7/NEDD4/LRRK2 axis. In terms of treatment of myocardial IRI, Katsur et al. showed that exosomes from NSCs could protect the heart by reducing infarct size while delaying cardiomyocyte mitochondrial permeability transition pore opening through gp130 signaling and downstream JAK/STAT pathway [130].

## The Hippo pathway regulated by SC-Exo therapy

The Hippo pathway is notable for their role in controlling organ size and regeneration, and its essential components and their functions are evolutionarily conserved [131]. Hippo pathway manifests as a kinase cascade including mammalian STE20-like kinase 1/2 (MST1/2), large tumor suppressor kinase 1/2 (LATS1/2) [132]. The upstream stimuli of Hippo cascade are exemplified by mechanical sensation, cell contact inhibition, polarity, energy status, stress, and hormonal factors. The two downstream effectors of Hippo cascade are Yes-associated protein 1 (YAP) and WW-domain-containing transcription regulator 1 (TAZ), which are transcriptional coactivators that regulate the expression of a broad spectrum of genes regulating apoptosis, cell proliferation, and stem cell self-renewal [133]. Therapeutic approaches targeting dysregulated Hippo pathway have shown potential in the treatment of a wide range of diseases [134].

In terms of fracture treatment, Yu et al. first developed a collagen HG-assisted 3D culture system to increase the yield and enhance the therapeutic effects of periodontal ligament stem cells-derived exosomes [135]. The underlying mechanism of better exosomal osteoinductivity in rats with bone defects is upregulation of the YAP signaling and Runx2/OPN activation. In terms of treatment of osteoporosis, Yang et al. used human UC MSC-derived exosomes via systemic injection [136]. Exosomal miR-1263 prevented apoptosis of BM MSCs in vitro and ameliorated disuse osteoporosis in rats. The exerted functions were achieved through binding of miRNA to the 3’untranslated region of Mob1 in recipient cells via Hippo pathway. In terms of treatment of premature ovarian insufficiency (POI), Li et al. demonstrated that UC MSC-derived exosomes could improve ovarian function and proliferation in CTX-induced POI mice [137]. These effects were achieved through Hippo pathway and reversed by a YAP inhibitor.

## The Notch pathway regulated by SC-Exo therapy

The Notch signaling is also an evolutionarily conserved pathway that possess a simple yet versatile mode of action. The canonical Notch pathway manifests as a cascade of proteolytic cleavage to release an intracellular domain (i.e., NICD) that serves to manipulate transcription [138]. The Notch pathway coordinates juxtacrine cellular signaling in which both the signal sending and receiving cells communicate through ligand-receptor crosstalk by which a wide range of cell fate decisions in cardiac, neuronal, and immune systems are determined [139]. Notch pathway is implicated in numerous contexts, such as organ development and repair, non-cancerous disease (e.g., OA, allergic asthma, graft versus host disease), and cancers [140]. More interestingly, Notch signaling controls the proliferation and differentiation of many types of stem cells, including embryonic stem cells (ESCs), NSCs, hematopoietic stem cells (HSCs), and Lgr5^+^ epithelial stem cells such as those found in the inner ear [141].

In terms of fracture treatment, Zhang et al. used a HA HG with nano-hydroxyapatite/poly-ε-caprolactone (PCL) scaffold for local delivery of UC MSC-derived exosomes [142]. In vitro, this combination enhanced the proliferation, migration, and angiogenic differentiation of endothelial stem/progenitor cells (EPCs) without negatively affecting the osteogenic potential of BM MSCs. In vivo, the exosomal miR-21 accelerated bone repair by boosting angiogenesis via the NOTCH1/DLL4 pathway. In terms of treatment of mechanical allodynia, Li et al. proved that exosomal miR-150-5p sourced from BM MSCs could suppress apoptosis and inflammation in the spinal dorsal horn [143]. Intrathecal injection of SC-Exo could alleviate mechanical allodynia in a rat model of L5 spinal nerve ligation by targeting NOTCH2 signaling in microglial cells.

## Minor signaling pathways regulated by SC-Exo therapy

Apart from the nine signaling pathways mentioned above, there are a dozen of other minor signaling pathways activated and regulated by stem cell-derived exosome-based treatment (Table 2). In orthopedic surgery, most relevant studies used BM MSC-derived exosomes delivered systemically. The affected signaling pathways include BMP-2/Smad1/RUNX2 signaling [144], SMURF1/RUNX2 signaling [145], and Angiopoietin-1/Tie2-NO signaling [146] during fracture treatment, c-MYC signaling [147] during OA treatment, and HIPK2/p53 signaling [148] during treatment of IDD.

**Table 2:** Minor signaling pathways influenced by stem cell-derived exosome-based therapy

Signaling pathway	Target disease	Source of exosome	Modification of exosome	Delivery modality	Essential findings	Refs
BMP-2/Smad1/RUNX2	fracture	BM MSC	N/A	systemic	enhanced osteogenesis and angiogenesis in rats	[144]
SMURF1/RUNX2	fracture	BM MSC	N/A	systemic	exosomal miR-25 regulated ubiquitination to promote fracture healing in mice	[145]
Angiopoietin-1/Tie2-NO	fracture	BM MSC	N/A	systemic	exosomal lncRNA-H19 promoted osteogenesis and angiogenesis in CBS-heterozygous mice	[146]
c-MYC	OA	BM MSC	N/A	systemic	modulated the level of chondrocyte glutamine metabolism by regulating c-MYC, thereby alleviating OA in rats	[147]
HIPK2/p53	IDD	BM MSC	N/A	local	exosomal miR-3594-5p prevented senescence in nucleus pulposus cells for disc regeneration in rats	[148]
NLRP3 inflammasome	SCI	epidural fat MSC	N/A	systemic	improved neurological functional recovery and reduced lesion volume	[149]
TSG-6/NF-κB/NLRP3	SNI	BM MSC	LPS precondition	systemic	accelerated peripheral nerve regeneration via M2 macrophage polarization	[150]
NLRP3 inflammasome	ischemic stroke	BM MSC	hypoxia precondition	systemic	rescued OGD-induced injury in rat neurons by suppressing NLRP3 inflammasome-mediated pyroptosis	[151]
caspase 2	ischemic stroke	NSC	N/A	systemic	exosomal miR-150-3p enhanced neuroprotective effects by targeting CASP2 in rats	[152]
SIRT1/HDAC	ischemic stroke	NSC	TSG101 oe	systemic	neuroprotective via anti-inflammatory activities, DNA damage pathway inhibition, and growth/trophic factor induction in rats	[40]
SIRT1-related	AD	NSC	N/A	systemic	enhanced mitochondrial function, synaptic activity, decreased inflammation, and rescued cognitive deficits in mice	[153]
Hand2/Phox2b	spina bifida aperta	NSC	N/A	systemic	exosomal Netrin 1 promoted neuronal differentiation of MSCs & NSCs in rats	[154]
CREB/BDNF/TrkB	brain ageing	NSC	N/A	systemic	counteracted HFD-induced memory impairment by modulating the CREB-dependent expression of synaptic plasticity-related genes	[155]
Akt/eNOS	wound healing	BM MSC	educated by neonatal serum	systemic	accelerated cutaneous wound healing via promoting angiogenesis in mice	[156]
Akt/eNOS	wound healing	BM MSC	atorvastatin-treated	systemic	exosomal miR-221-3p facilitated wound repair by enhancing angiogenesis in rats with streptozotocin-induced diabetic wound	[157]
CHK2/Beclin2	myocardial IRI	BM MSC	N/A	systemic	exosomal miR-143-3p reduced cell apoptosis and myocardial IRI by regulating autophagy in rats	[158]
Akt/ERK/AMPK	TMJ OA	MSC	N/A	systemic	promoted TMJ repair in immunocompetent rabbits by attenuating inflammation and restoring matrix homeostasis	[159]
Th1	corneal allograft rejection	BM MSC	N/A	systemic	crossed biological barrier and prolonged graft survival time in rats by inhibiting infiltration of CD4^+^ and CD25^+^ T cells	[160]
RANKL/RANK/OPG	periodontitis	BM MSC	N/A	local	promoted the regeneration of periodontal tissues, modulated immune-inflammatory response in rats	[161]

(AD = Alzheimer’s disease, akt = protein kinase B, AMPK = AMP-activated protein kinase, BDNF = brain-derived neurotrophic factor, BM = bone marrow, BMP = bone morphogenetic protein, CASP2 = caspase 2, CBS = cystathionine β-synthase, CHK2 = checkpoint kinase 2, CREB = cAMP response element binding, eNOS = endothelial nitric oxide synthase, ERK = extracellular signal-regulated kinase, exo = exosome, Hand2 = heart and neural crest derivatives expressed 2, HDAC = histone deacetylase, HFD = high fat diet, HIPK2 = homeodomain-interacting protein kinase 2, IDD = intervertebral disc degeneration, IRI = ischemia reperfusion injury, lncRNA = long non-coding RNA, LPS = lipopolysaccharide, miR = microRNA, MSC = mesenchymal stem cell, NF-κB = nuclear factor-kappa B, NLRP3 = NOD-, LRR- and pyrin domain-containing protein 3, NO = nitric oxide, NSC = neural stem cell, OA = osteoarthritis, oe = overexpressing, OGD = oxygen- & glucose-deprived, OPG = osteoprotegerin, Phox2b = paired like homeobox 2b, RANK = receptor activator of NF-κB, RANKL = RANK ligand, RUNX = runt-related transcription factor, SC = stem cell, SCI = spinal cord injury, SIRT1 = silent information regulator sirtuin 1, SNI = sciatic nerve injury, TMJ = temporomandibular joint, TrkB = thymosin receptor kinase-B, TSG-6 = tumor necrosis factor-stimulated gene-6, TSG101 = tumor suppressor gene 101)

In comparison, most relevant studies delivered NSC-derived exosomes via systemic administration in neurosurgery. These indications include SCI (e.g., NLRP3 inflammasome cascade [149]), SNI (e.g., TSG-6/NF-κB/NLRP3 cascade [150]), ischemic stroke (e.g., NLRP3 inflammasome cascade [151], caspase-2 signaling [152], SIRT1/HDAC signaling [40]), Alzheimer’s disease (AD) (e.g., SIRT1-related cascade [153]), spina bifida aperta (e.g., Hand2/Phox2b signaling [154]), and brain ageing (e.g., CREB/BDNF/TrkB signaling [155]).

In addition to orthopedic and neurosurgical diseases, other disorders have been experimented on through manipulation of minor signaling pathways. These include wound healing (e.g., Akt/eNOS signaling [156, 157]), myocardial IRI (e.g., CHK2/Beclin2 signaling [158]), OA in the temporomandibular joint (TMJ) (e.g., Akt/EKR/AMPK signaling [159]), corneal allograft rejection (e.g., Th1 signaling [160]), and periodontitis (e.g., RANKL/RANK/OPG signaling [161]). Their therapeutic effects were mostly achieved through systemically administered BM MSC-derived exosomes.

## Interactions between different signaling pathways regulated by SC-Exo therapy

While this review has covered a broad range of pathways, more detailed studies on the interactions between different signaling pathways when exosomes are applied could yield deeper insights into their therapeutic modulation. In other words, understanding how manipulating one pathway might affect others can provide insights into potential synergistic or antagonistic effects in therapeutic settings. However, there is only few relevant studies that qualify the above-mentioned objectives. Here, we attempt to provide a deeper exploration of the crosstalk between the highlighted pathways.

Firstly, in a traumatic SCI model, hypoxia preconditioning was found to be a promising and effective strategy to maximize the therapeutic effects of MSC-derived exosomes [36]. A miRNA array suggested miR-216a-5p to be the most enriched exosomal cargo. TLR4 was identified as the target downstream gene and the miR-216a-5p/TLR4/NF-κB axis was confirmed by a series of gain- and loss-of-function experiments. Through intermediation by exosomal miR-216a-5p, the PI3K/Akt signaling pathway was also involved in the modulation of microglial M1/M2 polarization. Secondly, in a cellular study, adipose MSC-derived exosomes might represent a promising approach for the treatment of neural injury [162]. These exosomes could suppress the cytotoxicity of activated microglia and the production of inflammatory cytokines by simultaneously inhibiting NF-κB and MAPK pathways. Finally, in a preclinical systemic lupus erythematosus model, MSC exosomal tsRNA-21,109 could inhibit macrophage M1 polarization through manipulation of various inflammation-related cascades such as Hippo, Wnt, MAPK and TGF-β signaling pathways [163].

## Preclinical considerations of SC-Exo therapy

## Limitations and challenges of SC-Exo therapy

Although the positive results of SC-Exo therapy have been highlighted in the above sections, discussions on the limitations, challenges, and instances where SC-Exo therapy did not perform as expected would provide a more realistic view of the current state of research. These are exemplified in three aspects: low yield, challenging extraction, and long-term effects.

Firstly, the relatively low yield and efficiency of exosomes have become one of the major obstacles preventing exosome therapy from entering clinical practice. For example, 1 ml cell culture medium can produce only less than 1 µg exosomal protein in a laboratory setting [164]. Various approaches of upscaling production of exosomes are available, including physical methods (starvation, hypoxia, and thermal stress), biochemical methods (e.g., BMP-2, HIF-1α, LPS, and TNF-α), instrumental methods (stirred tank bioreactors and hollow-fiber bioreactors) and mechanical methods (shear stress and 3D culturing) [165].

Secondly, the extraction and isolation of exosomes are relatively difficult to be standardized due to exosomal heterogeneity in size, content, surface markers, and source. The currently available techniques are based on exosomal size, surface charge, or immunoaffinity [166]. However, there is no ‘one-fits-all’ approach as these strategies all have pros and cons. Variability in these processes can impact the purity and integrity of exosomes, thereby affecting therapeutic outcomes. For example, although ultracentrifugation is considered the gold standard for exosome extraction as it needs minimal consumables and expertise, the time consumption, low efficiency and high cost have limited its large-scale use [167]. Likewise, although size-based isolation methods (e.g., ultrafiltration and size-exclusion chromatography) are fast and appropriate for large-scale applications, low purity, exosome loss, and pore clogging are making them difficult to be routinely used [168]. Therefore, combining these methods (e.g., microfluidics-based with precipitation-based ones) might be a solution to satisfy multiple requirements for exosome extraction and isolation.

Lastly, few longitudinal studies tracked the long-term effects and stability of therapeutic outcomes post-exosome treatment. Most of them used MSC-derived exosomes targeting neurosurgical diseases. For example, in a stroke preclinical experiment, various aspects such as motor coordination deficits, histological brain injury, immune responses in the peripheral blood and brain, and cerebral angiogenesis and neurogenesis were all improved for as long as 28 days after stroke [169]. In addition, MSC-Exo could suppress iNOS production and correct neural impairment in AD mice [170], as well as inhibit inflammation and prevent abnormal neurogenesis after status epilepticus [171], both of which lead to long-term cognitive and memory improvement. Finally, in a swine TBI model complicated by hemorrhagic shock, MSC-Exo could provide neuroprotection and enhance long-term neurologic outcomes [172]. The long-term therapeutic effects of exosomes might be preserved using various storage methods such as cryopreservation, lyophilization, and spray-drying [173]. Specifically, the choice of antifreeze and preserving temperature are two determining factors. For example, non-permeable disaccharide antifreeze (e.g., trehalose) excels as it prevents exosome aggregation and cryodamage [174]. Storage of exosomes at -80^o^C is considered a suitable temperature with least impact on exosome morphology and cargo, whereas 4^o^C might diminish the biological activity and weaken the protein content of exosomes [175, 176].

## Therapeutic comparison of SC-Exo

Almost all types of human cells can generate exosomes. In addition, most types of stem cells can produce exosomes with therapeutic potential. Currently, exosomes are classified mainly according to the type of their parental cells. These include, but are not limited to, ESCs, iPSCs, (two best examples of pluripotent stem cells), HSCs, MSCs, NSCs, and EPCs (examples of adult multipotent stem cells [14]. Although there is no single study directly comparing exosomes from different stem cell sources in terms of their therapeutic effectiveness for a given disease, cross-study and qualitative comparison could be made. A discussion on whether different SC-Exo have unique benefits or drawbacks would be valuable for understanding their potential therapeutic applications.

For example, both MSC- and NSC-derived exosomes have demonstrated considerable effects in preclinical experiments for SCI treatment [93]. However, MSC-derived exosomes tended to manifest more anti-inflammatory and immunomodulatory effects [36, 78, 149], whereas NSC-derived ones exhibited more neuronal protection and microvascular regeneration [61, 177, 178]. Another example is the treatment of cardiothoracic conditions in which ESC- and MSC-derived exosomes showed comparable therapeutic effects. These effects are exemplified by protection of cardiomyocytes, cardiac remodeling, and attenuation of heart failure [93]. In short, more in-depth and comparative studies are required to determine which cell types yield the most therapeutically potent exosomes. Variables like tissue source, exosome concentration, cargo identification, preconditioning, exosome uptake, and therapeutic longevity all need to be considered before a reliable comparison can be made.

## Translation of preclinical studies into clinical trials

A search on ClinicalTrials.gov using ‘exosome therapy’, ‘exosome treatment’, and ‘exosome’ as keywords generated 60 records that directly use exosomes as therapeutic agents [93]. These 60 clinical trials represent the latest achievement of how SC-Exo therapy can be translated into clinical practice. On one hand, the spectrum of diseases (i.e., medical and surgical conditions) covered is very extensive. These include many surgical disorders discussed in Sects. 2 to 11, such as orthopedic, neurosurgical, plastic surgical, general surgical, cardiothoracic, and ophthalmology diseases. However, preclinical studies targeting many other diseases have not yet been developed into clinical trials. On the other hand, most of the qualified trials used MSC as the cellular source for exosomes, which differs significantly from the preclinical studies discussed in Sects. 2 to 11. The unpopularity of NSC-derived exosomes in clinical trials might be partially due to supply constraints of their parental cells [179]. As one of the solutions, fibroblast-derived iNSCs have created a brilliant opportunity for obtaining exosomes from NSC-like cells [180, 181].

## Conclusion and future perspectives

Stem cell-derived exosomes inherit similar therapeutic advantages from their parental stem cells without exhibiting immune, tumorigenic, and ethical complications. The therapeutic effects exerted by SC-Exo during the treatment for various diseases are hierarchically magnified all the way through molecular level (e.g., signaling cascades), cellular level (e.g., cell proliferation and differentiation), and tissue level (e.g., regeneration and anti-inflammation). As the most fundamental aspect of SC-Exo therapy, a wide range of signaling cascades activated and regulated by exosomes are exemplified by major pathways (e.g., the PTEN/PI3K/Akt/mTOR, NF-κB, TGF-β, HIF-1α, Wnt, MAPK, JAK-STAT, Hippo, and Notch signaling cascades) and minor pathways. In addition, there are extensive crosstalk among these signaling pathways. As a result, SC-Exo therapy has exhibited promising results in managing numerous diseases, such as those in in orthopedic surgery (e.g., fracture, osteoarthritis), neurosurgery (e.g., traumatic brain injury, ischemic stroke), cardiothoracic surgery (e.g., ischemic heart disease, myocardial infarction), plastic surgery (e.g., wound healing), general surgery (e.g., liver fibrosis), etc. In conclusion, exploring and understanding context-specific molecular mechanism of SC-Exo therapy help fuel and guide further preclinical studies and clinical trials. Future targets in this intriguing field should focus on verifying other signaling pathways (e.g., Hedgehog cascade, RTK cascade), modifying natural exosomes (e.g., pre-isolation and post-isolation), and upscaling the yield of exosomes (e.g., biochemical, physical, and mechanical strategies).

## Data Availability

Available upon request.
